# Psychosocial Well-Being at the Time of Trauma Exposure and Risk of PTSD

**DOI:** 10.1001/jamanetworkopen.2024.40388

**Published:** 2024-10-25

**Authors:** Dawne Vogt, Shelby Borowski, Shaina A. Kumar, Lewina O. Lee, Paula P. Schnurr

**Affiliations:** 1 Women’s Health Sciences Division, National Center for PTSD, VA Boston Healthcare System, Boston, Massachusetts; 2 Department of Psychiatry, Boston University Chobanian & Avedisian School of Medicine, Boston, Massachusetts; 3 Behavioral Science Division, National Center for PTSD, VA Boston Healthcare System, Boston, Massachusetts; 4 Executive Division, National Center for PTSD, VA Boston Healthcare System, Boston, Massachusetts; 5 Department of Psychiatry, Geisel School of Medicine, Hanover, New Hampshire

## Abstract

This cohort study examines whether 3 well-being domains—vocational, financial, and social—are associated with the risk of developing subsequent posttraumatic stress disorder (PTSD) after a traumatic event.

## Introduction

Given that most individuals will experience at least 1 traumatic event in their lifetime, it is critical to identify factors that protect against negative mental health consequences, such as posttraumatic stress disorder (PTSD). Although some studies have examined how pretrauma factors affect PTSD,^1,2^ most research has examined risk rather than protective factors. Here, we investigated whether individuals with higher pretrauma vocational well-being (V-WB), financial well-being (F-WB), and social well-being (S-WB) were less likely to experience PTSD following traumatic events, building on the expectation that these individuals may have better access to resources that promote coping and trauma recovery. The resulting knowledge can inform efforts to enhance resilience to PTSD in populations at high risk for trauma exposure, including those who serve in the military, the current study focus.

## Methods

Data for this cohort study were from the Veterans Metrics Initiative Study, a large prospective national study of US military veterans who completed biannual surveys during the first 3 years after discharge (2016-2019).^3^ Participants reported on their V-WB, F-WB, and S-WB on the Well-Being Inventory, a validated measure of psychosocial well-being.^4^ Their experiences of probable PTSD (P-PTSD), prior trauma exposure, educational attainment, race and ethnicity, stress exposure, probable depression, and preexisting P-PTSD were also assessed. We obtained institutional review board approval from the VA Boston Healthcare System and informed consent. We followed STROBE reporting guidelines, drawing data from 4 time points (T) that used the same P-PTSD measure (approximately 15 to approximately 33 months after discharge) in 2024. We applied modified Poisson regressions with robust error variances to examine the prospective association of pretrauma well-being with P-PTSD at the subsequent surveys among those who reported trauma exposure between each time frame. All models adjusted for prior trauma exposure. Additional models were implemented to evaluate potential confounders, and a final model included all well-being domains to evaluate their unique contribution. Analyses were completed in Stata MP version 18.0 (StataCorp), and statistical significance was set at *P* < .05. Please see the eMethods in Supplement 1 for more details.

## Results

Table 1 displays sample characteristics in the 3 analytical time frames. The sample comprised 978 individuals (773 [79.0%] men; mean [SD] age, 33.0 [9.3] years).

**Table 1.** Sample Characteristics and Study Descriptives for the 3 Analytical Time Frames^a^

Characteristic	Participants, No. (%)		
	T3-T4 sample (n = 482)	T4-T5 sample (n = 377)	T5-T6 sample (n = 346)
Age at discharge, y			
<25	124 (25.8)	94 (24.9)	82 (23.7)
25 to <35	176 (36.6)	140 (37.2)	137 (39.6)
≥35	181 (37.6)	143 (37.9)	127 (36.7)
Race and ethnicity			
Black, non-Hispanic	63 (13.2)	41 (11.0)	63 (9.3)
Hispanic	82 (17.1)	54 (14.5)	58 (16.9)
White, non-Hispanic	288 (60.3)	237 (63.5)	219 (63.9)
Other race, non-Hispanic^b^	45 (9.4)	41 (11.0)	34 (9.9)
Gender			
Men	387 (80.3)	292 (77.5)	277 (80.1)
Women	95 (19.7)	85 (22.5)	69 (19.9)
Level of education at discharge			
Less than bachelor’s degree	346 (71.8)	253 (67.1)	249 (72.0)
Bachelor’s degree or higher	136 (28.2)	124 (32.9)	97 (28.0)
Subsequent PTSD			
No probable PTSD	272 (56.4)	227 (60.2)	191 (55.2)
Probable PTSD	210 (43.6)	150 (39.8)	155 (44.8)
Prior trauma exposure			
No prior trauma exposure	39 (8.1)	38 (10.1)	23 (6.7)
Prior trauma exposure	443 (91.9)	339 (89.9)	323 (93.3)
Prior stress exposure	13.9 (8.6)	12.6 (8.6)	13.8 (8.6)
Prior probable depression			
No probable depression	340 (70.5)	254 (67.5)	230 (66.7)
Probable depression	142 (29.5)	122 (32.5)	115 (33.3)
Prior probable PTSD			
No probable PTSD	296 (61.5)	236 (63.4)	203 (58.8)
Probable PTSD	185 (38.5)	136 (36.6)	142 (41.2)

Abbreviations: PTSD, posttraumatic stress disorder; T, time point.

^a^ Samples were not mutually exclusive, as some participants reported a new trauma at multiple time points. Prior indicates that this characteristic was measured at the first point within the analytical time frame (eg, refers to T3 within T3-T4 time frame). Subsequent indicates that this characteristic was measured at the second point within the analytical time frame (eg, refers to T4 within the T3-T4 time frame). All variables were self-reported.

^b^ Other race, non-Hispanic includes American Indian or Alaska Native, Asian, multiracial, and Native Hawaiian or Other Pacific Islander individuals.

Both overall and domain-based measures of well-being were associated with lower risk of P-PTSD in the T3 to T4 time frame after adjusting for prior trauma exposure. Analyses of associations in the T4 to T5 and T5 to T6 time frames largely replicated these findings (Table 2). Nearly all associations between overall and domain-specific measures of well-being and P-PTSD remained significant and similar in magnitude when accounting for education, race and ethnicity, and stress exposure. After adjusting for probable depression, overall well-being associations remained significant, and all domain-based measures were associated with P-PTSD in at least 2 time frames. The same held true when controlling for prior P-PTSD, except that F-WB was no longer associated with P-PTSD. Both S-WB and V-WB maintained unique associations with P-PTSD in models with all domains included, but F-WB was not associated with P-PTSD in 2 of 3 time frames.

**Table 2.** Well-Being and Subsequent PTSD Among Veterans With a New Trauma Exposure, Adjusted for Prior Trauma Exposure and Additional Covariates

Model^a^	RR (95% CI)		
	Time 4 PTSD	Time 5 PTSD	Time 6 PTSD
Controlling for prior trauma			
Model 1: prior overall WB	0.37 (0.27-0.52)^b^	0.38 (0.26-0.55)^b^	0.39 (0.26-0.58)^b^
Model 2: prior social WB	0.38 (0.27-0.53)^b^	0.46 (0.33-0.66)^b^	0.37 (0.25-0.56)^b^
Model 3: prior financial WB	0.52 (0.37-0.72)^b^	0.53 (0.36-0.77)^b^	0.60 (0.40-0.81)^b^
Model 4: prior vocational WB	0.57 (0.46-0.71)^b^	0.49 (0.38-0.64)^b^	0.59 (0.46-0.77)^b^
Controlling for prior trauma, education, and race and ethnicity			
Model 1: prior overall WB	0.40 (0.28-0.56)^b^	0.40 (0.28-0.59)^b^	0.41 (0.28-0.62)^b^
Model 2: prior social WB	0.38 (0.27-0.54)^b^	0.50 (0.35-0.72)^b^	0.39 (0.26-0.59)^b^
Model 3: prior financial WB	0.57 (0.41-0.79)^b^	0.57 (0.39-0.85)^b^	0.66 (0.44-0.99)^b^
Model 4: prior vocational WB	0.58 (0.47-0.72)^b^	0.52 (0.40-0.68)^b^	0.60 (0.47-0.78)^b^
Controlling for prior trauma and stress exposure			
Model 1: prior overall WB	0.49 (0.34-0.70)^b^	0.50 (0.34-0.73)^b^	0.50 (0.34-0.75)^b^
Model 2: prior social WB	0.48 (0.34-0.67)^b^	0.62 (0.43-0.90)^b^	0.49 (0.32-0.74)^b^
Model 3: prior financial WB	0.72 (0.51-1.01)	0.72 (0.48-1.06)	0.80 (0.54-1.18)
Model 4: prior vocational WB	0.67 (0.54-0.82)^b^	0.60 (0.46-0.78)^b^	0.73 (0.56-0.95)^b^
Controlling for prior trauma and depression			
Model 1: prior overall WB	0.52 (0.37-0.74)^b^	0.60 (0.42-0.86)^b^	0.54 (0.37-0.80)^b^
Model 2: prior social WB	0.53 (0.37-0.76)^b^	0.75 (0.54-1.04)	0.49 (0.33-0.72)^b^
Model 3: prior financial WB	0.66 (0.49-0.89)^b^	0.72 (0.52-0.99)^b^	0.82 (0.56-1.19)
Model 4: prior vocational WB	0.76 (0.62-0.93)^b^	0.70 (0.55-0.90)^b^	0.76 (0.59-0.97)^b^
Controlling for prior trauma and PTSD			
Model 1: prior overall WB	0.61 (0.45-0.84)^b^	0.66 (0.47-0.92)^b^	0.68 (0.47-0.98)^b^
Model 2: prior social WB	0.69 (0.49-0.96)^b^	0.80 (0.58-1.10)	0.55 (0.38-0.81)^b^
Model 3: prior financial WB	0.79 (0.60-1.04)	0.77 (0.57-1.06)	1.01 (0.72-1.42)
Model 4: prior vocational WB	0.83 (0.70-0.99)^b^	0.75 (0.60-0.95)^b^	0.84 (0.67-1.05)
Independent associations of WB domains controlling for prior trauma			
Prior social WB	0.45 (0.32-0.64)^b^	0.59 (0.41-0.84)^b^	0.43 (0.28-0.66)^b^
Prior financial WB	0.71 (0.52-0.99)^b^	0.71 (0.48-1.05)	0.86 (0.59-1.25)
Prior vocational WB	0.69 (0.56-0.85)^b^	0.59 (0.45-0.77)^b^	0.72 (0.55-0.93)^b^

Abbreviations: PTSD, posttraumatic stress disorder; RR, risk ratio; WB, well-being.

^a^ Prior indicates that the characteristic was measured at time point prior to PTSD measurement. In models labeled 1 to 4, only 1 WB factor was entered in the model at a time. In the model examining independent associations of WB domains, all 3 WB domains were entered together in a single model. Some individuals reported trauma at multiple time points and were included in more than 1 time frame in replication analyses. To evaluate whether this might have introduced bias, sensitivity analyses were conducted that removed individuals with multiple reports of trauma, so they were only included in the first time frame that they reported trauma. Because results remained largely similar and the interpretation of findings did not change, the more fully powered analyses appear in this table.

^b^ *P* < .05.

## Discussion

Results provide evidence for the protective role that pretrauma psychosocial well-being may offer in reducing risk for PTSD, supporting the call for greater investment in well-being promotion among populations at risk for poor health^5,6^ and highlighting the value of focusing these efforts on the extent to which individuals are doing well in key life roles and activities. These findings also extend research demonstrating the impact of social factors on PTSD (eg, social support) and underscore the need for greater attention to how success in vocational pursuits affects experiences of PTSD. They also provide guidance regarding how best to prioritize the provision of psychosocial well-being programming, pointing to the particular value that interventions that can improve relationship quality and vocational outcomes may offer in PTSD risk reduction. Limitations of the study include reliance on self-reported PTSD measurement and use of an observational rather than experimental design; nonetheless, confidence in the protective role of psychosocial well-being is increased by our replication of findings and evaluation of potential confounders.

## Notes

Supplement 1.
eMethods.

eReferences.

Supplement 2.
Data Sharing Statement
