# Supplementary material for: Psychosocial Well-Being at the Time of Trauma Exposure and Risk of PTSD
Source: JAMA Netw Open. 2024 Oct 25;7(10):e2440388. doi: 10.1001/jamanetworkopen.2024.40388 (PMC11512332; doi:10.1001/jamanetworkopen.2024.40388)
Supplement: Supplement 1. — eMethods. eReferences. [file jamanetwopen-e2440388-s001.pdf]

Supplemental Online Content

Vogt D, Borowski S, Kumar SA, Lee LO, Schnurr PP. Psychosocial well-being at the time of trauma exposure and risk of PTSD. *JAMA Netw Open.* 2024;7(10):e2440388. doi:10.1001/jamanetworkopen.2024.40388

eMethods.

eReferences.

This supplemental material has been provided by the authors to give readers additional information about their work.

© 2024 Vogt D et al. *JAMA Network Open.*

eMethods

TVMI Study and Sample

The current analyses drew from the Veterans Metrics Initiative (TVMI) Study, a large prospective cohort study of a national sample of newly separated U.S. military veterans identified from a list of all separating service members. Participants completed six assessments at six-month intervals beginning within approximately three months after military discharge. For the purpose of this analysis, which was to examine the relationship between pre-trauma well-being and subsequent risk for PTSD among individuals who experienced trauma during the study period, we limited the analytical sample to individuals who reported a trauma exposure during at least one of the three timeframes included in analyses (N= 978). Analyses drew from Time 3 (T3, ~15 months post-discharge), Time 4 (T4, ~21 months post-discharge), Time 5 (T5, ~27 months post-discharge), and Time 6 (T6, ~33 months post-discharge) data because these timepoints used the same PTSD measure.

Well-Being Measurement and Scoring

Veterans completed the Well-Being Inventory (WBI)[1] with respect to the three-month period preceding each timepoint. This measure assesses status, functioning, and satisfaction with regard to vocational, financial, and social life domains. Status indicators address whether individuals have a vocation (e.g., paid or volunteer work, school or training), a secure financial situation, and are partnered or have regular social contact with other community members. Functioning and satisfaction measures address how well veterans are able to fulfill key role functions (e.g., *completed work when expected, followed a budget, provided support or help to friends when needed*), as well as how satisfied they are with regard to each life domain (e.g., *how much work contributions are valued, amount of savings, sense of belonging in community*).

Functioning and satisfaction items were rated on a response format that ranged from 1 to 5. Functioning items response options were 1 = *Never* to 5 = *Most or all of the time.* Satisfaction item response options were 1 = *Very dissatisfied* to 5 = *Very satisfied.* Functioning and satisfaction items were averaged for each measure with an average possible score ranging from 1 to 5, with higher scores indicating better functioning or satisfaction. For the purposes of the coding used within this paper, participants with average scores ≥ 3.668 were considered to be functioning well and satisfied. Those individuals with average scores ≤ 3.667 were identified as not functioning well or satisfied.

Following procedures used in prior research,[2] for vocational well-being individuals received a score of 0 if they reported no participation in work or school, 1 if they had a vocation but were not functioning well or satisfied, 2 if they had a vocation and were either functioning well or satisfied, and 3 if they had a vocation and were both functioning well and satisfied. If in work and school, individuals needed to score above the threshold for both domains to be considered functioning well or satisfied within the vocational domain. Scores were then dichotomized into higher vocational well-being (scores of 3 were re-coded as 1) and lower vocational well-being (scores ≤ 2 were coded as 0).

© 2024 Vogt D et al. *JAMA Network Open.*

For financial well-being, individuals received a score of 3 if they had a secure financial status, were functioning well financially, and were satisfied with their financial situation, 2 if they endorsed any two of these circumstances, 1 if they endorsed only one of these circumstances, and 0 if they had a problematic/at-risk financial status, poor financial functioning, and low financial satisfaction. Scores were then dichotomized into higher financial well-being (scores of 3 were re-coded as 1) and lower financial well Individuals with lower financial well-being (scores ≤ 2 were re-coded as 0).

For social well-being individuals received a score of 0 if they reported no involvement in an intimate relationship or regular contact with friends/extended family/community, 1 if they had social involvement, but were not functioning well or satisfied, 2 if they had social involvement and were either functioning well or satisfied, and 3 if they had social involvement and were both functioning well and satisfied. Similar to vocational scoring, if the individual was in an intimate relationship and had broader community involvement they needed to score above the threshold for both domains to be considered functioning well or satisfied within the social domain. Scores were then dichotomized into higher social well-being (scores of 3 were re-coded as 1) and lower social well-being (scores ≤ 2 were re-coded as 0).

To create overall well-being scores interim vocational, financial, and social domain scores were averaged. Individuals with missing data for any domain did not receive an overall well-being score. Scores ranged from 0 to 3 with higher scores indicating higher well-being. Scores were then dichotomized into higher overall well-being (scores > 2 were re-coded as 1) and lower overall well-being (scores ≤ 2 were re-coded as 0).

The WBI has been validated in prior research.[1] Cronbach's alphas were satisfactory, with an average alpha of 0.86. The majority of WBI measure discriminated among individuals with and without mental health conditions and demonstrated expected declines among those with a new mental health condition. In addition, confirmatory factor analyses supported the proposed factor structure for the WBI functioning and satisfaction measures.

<u>PTSD Measurement and Scoring</u>

The abbreviated 8-item PCL-5 measure was used to assess probable PTSD, which correlates highly with the full PCL-5.[3] A cut-off score of 12 or higher was used to classify respondents as having probable PTSD (0 = no probable PTSD; 1 = probable PTSD).[4]

<u>Measurement of Covariates</u>

The following measures were used to assess covariates: (1) prior trauma exposure (PC-PTSD-5 lead-in question, also used to identify participants with trauma exposure for inclusion in analyses; 0 = no prior trauma exposure; 1 = had prior trauma exposure)[5,6]; (2) T1 educational attainment (possible scores range from 1 = high school degree/GED to 6 = advanced professional degree) and racial/ethnic status (0 = White, Non-Hispanic; 1= Black, Non-Hispanic; 2 = Other race(s), Non-Hispanic; 3 = Hispanic); (3) T3-T5 stress exposure, as assessed by a 13-item

© 2024 Vogt D et al. *JAMA Network Open.*

stressor index with possible scores from 0 to 52, with higher scores indicating higher levels of stress exposure; (4) probable depression, as assessed by the Patient Health Questionnaire-2 (PHQ-2; 0 = no probable depression; 1 = probable depression)[7]; and (5) prior probable PTSD, assessed by the abbreviated PCL-5 using a cut-off score of 12 or higher to classify respondents as having probable PTSD (scored as 0 for no trauma individuals; 0 = no prior probable PTSD for those with a trauma; 1 = prior probable PTSD).[3]

Representativeness of Sample

Although we drew our sample for this study from a population-based sampling frame it is possible that non-response at either the item or unit level could have biased results. To evaluate this possibility, we examined whether either type of missing data was a substantial problem for the study. Our examination of item-level missingness revealed very little missing data, aside from cases in which individuals were directed not to answer questions that were not relevant to them (e.g., questions about intimate relationship functioning for those who did not report being in a relationship). Specifically, item level missingness ranged from 0% to 1.8% for well-being and PTSD measures. Thus, we determined that item non-response was not a substantial concern in this study, and it was not necessarily to adjust for this type of missingness in the current study.

Given that unit-level missingness, as reflected in differential participation or drop-out from the study, may also bias study findings we next compared how representative the 23% of veterans who elected to enroll in the study were relative to the larger population-based sampling frame from which the sample was drawn. These findings revealed that respondents were similar to the sampling frame on many characteristics (e.g., gender, race/ethnicity, branch of service), although lower enlisted service members were somewhat less likely to participate than officers. We also compared veterans who dropped out at each timepoint to those who did not drop out on a wide variety of study variables (demographic, military, health, work and financial characteristics) that were assessed at T1. Few differences emerged for these comparisons with the exception that veterans who identified as warrant officers (compared to enlisted personnel), endorsed poorer initial financial status, reported lower education, and gave an initial mailing address that was not geocodable (“bad” addresses) were somewhat less likely to participate at later timepoints. However, meaningful differences did not emerge on other characteristics, including health measures.

© 2024 Vogt D et al. *JAMA Network Open.*

eReferences

1. Vogt D, Taverna EC, Nillni YI, et al. Development and validation of a tool to assess military veterans' status, functioning, and satisfaction with key aspects of their lives. *Appl Psychol*. 2019;11(2):328-349. doi:10.1111/aphw.12161
2. Vogt, D., King, M. W., Borowski, S., Finley, E. P., Perkins, D. F. & Copeland, L. A. (2021). Identifying factors that contribute to military veterans' post-military well-being. *Applied Psychology: Health and Well-Being. 13*, 341-356. doi:10.1111/aphw.12252
3. Geier TJ, Hunt JC, Hanson JL, et al. Validation of abbreviated four-and eight-item versions of the PTSD checklist for DSM-5 in a traumatically injured sample. *J Trauma Stress*. 2020;33(3):218-226. doi:10.1002/jts.22478
4. Price M, Szafranski DD, van Stolk-Cooke K, Gros DF. Investigation of abbreviated 4 and 8 item versions of the PTSD Checklist 5. *Psychiatry Res*. 2016;239:124-130. doi:10.1016/j.psychres.2016.03.014
5. Geier TJ, Hunt JC, Hanson JL, et al. Validation of abbreviated four-and eight-item versions of the PTSD checklist for DSM-5 in a traumatically injured sample. *J Trauma Stress*. 2020;33(3):218-226. doi:10.1002/jts.22478
6. Prins A, Ouimette P, Kimerling R, et al. The primary care PTSD screen (PC-PTSD): Development and operating characteristics. *Primary Care Psychiatry*. 2003;9(1):9-14. doi:10.1185/135525703125002360
7. Bovin MJ, Kimerling R, Weathers FW, et al. Diagnostic accuracy and acceptability of the primary care posttraumatic stress disorder screen for the diagnostic and statistical manual of mental disorders among US veterans. *JAMA Network Open*. 2021;4(2):e2036733-e2036733. doi:10.1001/jamanetworkopen.2020.36733

© 2024 Vogt D et al. *JAMA Network Open.*
