# Supplementary material for: Psychosocial Well-Being at the Time of Trauma Exposure and Risk of PTSD
Source: JAMA Netw Open. 2024 Oct 25;7(10):e2440388. doi: 10.1001/jamanetworkopen.2024.40388 (PMC11512332; doi:10.1001/jamanetworkopen.2024.40388)
Supplement: Supplement 2. — Data Sharing Statement [file jamanetwopen-e2440388-s002.pdf]

# Data Sharing Statement

Vogt. Psychosocial Well-Being at the Time of Trauma Exposure and Risk of PTSD. *JAMA Netw Open*. Published October 25, 2024. doi:10.1001/jamanetworkopen.2024.40388

**Data**
**Data available:** No

**Additional Information**
**Explanation for why data not available:** A deidentified dataset is already available online.
